# Clines, Clusters, and the Effect of Study Design on the Inference of Human Population Structure

**DOI:** 10.1371/journal.pgen.0010070

**Published:** 2005-12-09

**Authors:** Noah A Rosenberg, Saurabh Mahajan, Sohini Ramachandran, Chengfeng Zhao, Jonathan K Pritchard, Marcus W Feldman

**Affiliations:** 1 Department of Human Genetics, Bioinformatics Program, and the Life Sciences Institute, University of Michigan, Ann Arbor, Michigan, United States of America; 2 Department of Computer Science, University of Southern California, Los Angeles, California, United States of America; 3 Department of Biological Sciences, Stanford University, Stanford, California, United States of America; 4 Mammalian Genotyping Service, Marshfield Clinic Research Foundation, Marshfield, Wisconsin, United States of America; 5 Department of Human Genetics, University of Chicago, Chicago, Illinois, United States of America; University of Alabama at Birmingham, United States of America

## Abstract

Previously, we observed that without using prior information about individual sampling locations, a clustering algorithm applied to multilocus genotypes from worldwide human populations produced genetic clusters largely coincident with major geographic regions. It has been argued, however, that the degree of clustering is diminished by use of samples with greater uniformity in geographic distribution, and that the clusters we identified were a consequence of uneven sampling along genetic clines. Expanding our earlier dataset from 377 to 993 markers, we systematically examine the influence of several study design variables—sample size, number of loci, number of clusters, assumptions about correlations in allele frequencies across populations, and the geographic dispersion of the sample—on the “clusteredness” of individuals. With all other variables held constant, geographic dispersion is seen to have comparatively little effect on the degree of clustering. Examination of the relationship between genetic and geographic distance supports a view in which the clusters arise not as an artifact of the sampling scheme, but from small discontinuous jumps in genetic distance for most population pairs on opposite sides of geographic barriers, in comparison with genetic distance for pairs on the same side. Thus, analysis of the 993-locus dataset corroborates our earlier results: if enough markers are used with a sufficiently large worldwide sample, individuals can be partitioned into genetic clusters that match major geographic subdivisions of the globe, with some individuals from intermediate geographic locations having mixed membership in the clusters that correspond to neighboring regions.

## Synopsis

By helping to frame the ways in which human genetic variation is conceptualized, an understanding of the genetic structure of human populations can assist in inferring human evolutionary history, as well as in designing studies that search for disease-susceptibility loci. Previously, it has been observed that when individual genomes are clustered solely by genetic similarity, individuals sort into broad clusters that correspond to large geographic regions. It has also been seen that allele frequencies tend to vary continuously across geographic space. These two perspectives seem to be contradictory, but in this article the authors show that they are indeed compatible.

First the authors demonstrate that the clusters are robust, in that if sufficient data are used, the geographic distribution of the sampled individuals has little effect on the analysis. They then show that allele frequency differences generally increase gradually with geographic distance. However, small discontinuities occur as geographic barriers are crossed, allowing clusters to be produced. These results provide a greater understanding of the factors that generate the clusters, verifying that they arise from genuine features of the underlying pattern of human genetic variation, rather than as artifacts of uneven sampling along continuous gradients of allele frequencies.

## Introduction

It has recently been demonstrated in several studies that to a large extent, without prior knowledge of individual origins, the geographic ancestries of individuals can be inferred from genetic markers [1–5]. In one of the most extensive of these studies to date, considering 1,056 individuals from 52 human populations, with each individual genotyped for 377 autosomal microsatellite markers, we found that individuals could be partitioned into six main genetic clusters, five of which corresponded to Africa, Europe and the part of Asia south and west of the Himalayas, East Asia, Oceania, and the Americas [3]. Some individuals from boundary locations between these regions were inferred to have partial ancestry in the clusters that corresponded to both sides of the boundary. In many cases, subclusters that corresponded to individual populations or to subsets of populations were also identified.

To further ascertain the degree of difficulty in obtaining the genetic clusters, several articles have considered the influence of properties of the study design on the extent of clustering [3,4,6–10]. These studies have shown that the clustering patterns are robust, provided that at least about 60–150 markers are used [3,4,7,9], or about 40 or fewer if markers are preselected to have a high information content about ancestry [6]. They have also observed that although clustering patterns are influenced by sample size for small samples, the cluster membership estimates obtained for individuals in analysis of subsamples of larger datasets are close to those seen in analysis of the full data [9]. Additionally, they have found clustering results obtained with different statistical techniques to be quite similar [7,8].

Other factors besides sample size and number of markers, however, may influence clustering patterns. Serre and Pääbo [10] argued that the geographic dispersion of the sample and the assumption made about whether or not allele frequencies are correlated across populations had substantial influences on genetic clustering. They suggested that individuals are less strongly placed into clusters when the sample is more geographically uniform, and when allele frequencies are assumed to be uncorrelated. Consequently, they claimed that the geographic clusters obtained by Rosenberg et al. [3] were artifacts of the sampling design and of the use of a model of correlation among allele frequencies across populations. However, much of the geographic dispersion analysis of [10] was based on two datasets with 89 and 90 individuals and 20 loci, in general too little data for clustering to be apparent [3,4,9]. The remainder of their geographic analysis, as well as the source of their comments about uncorrelated frequencies, was a comparison to the Rosenberg et al. [3] results of several analyses of 261 individuals chosen to be equally distributed across the 52 populations studied. Serre and Pääbo's analyses assumed allele frequencies to be uncorrelated across populations, whereas Rosenberg et al. had assumed that they were correlated. Thus, although a difference in results was seen between the analyses in [10] and those in [3], the attribution of this difference specifically to a difference in geographic dispersion or to a difference in assumptions about allele frequency correlations is problematic, because both of these variables differed between studies, as did the number of individuals.

**Figure 1:**
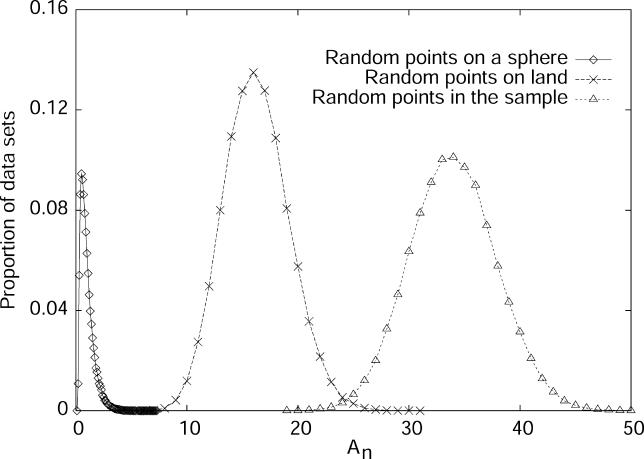
Distribution of the Geographic Dispersion Statistic *(A_n_)* for Sets of 100 Points Randomly Sampled from a Sphere, Randomly Sampled from the Land Area of the Earth (from among the Points Plotted in Figure 5 of [11]), and Randomly Sampled from the Reported Locations of Individuals in the Dataset Each distribution is obtained by binning the values of *A_n_* for 100,000 sets of points.

In this article, we perform an extensive evaluation of the role of study design on genetic clustering, considering both geographic dispersion and allele frequency correlation, as well as sample size, number of loci, and number of clusters. The dataset employed is an expansion of our original data [3] to 993 markers, including 783 microsatellites [11] and 210 insertion/deletion polymorphisms. Analysis of multilocus genotypes in the larger dataset reveals essentially the same set of clusters as was produced with the original 377 markers. The number of loci, sample size, and number of clusters are observed to have considerable influence on clustering. In agreement with the suggestion of [10], the assumption made about allele frequency correlations is also seen to have a substantial impact. Because large allele frequency correlations exist across populations, however, the basis for the supposition by [10] that allele frequencies are uncorrelated is questionable. Finally, the level of geographic dispersion of the sample is seen to have only a relatively small effect on the clustering results, and this variable is not consistent in the direction in which it influences the level of clustering. Therefore, we find no reason to interpret our inferred clusters as artifacts of the sampling design in our original study, and we conclude with an illustration of how the clusters can have arisen from small discontinuities in genetic distance across geographic barriers.

## Results

We utilized the unsupervised clustering algorithm implemented in STRUCTURE [12,13] to group individuals into genetic clusters in such a way that each individual is given an estimated membership coefficient for each cluster, corresponding to the fraction of his or her genome inferred to have ancestry in the cluster. This method requires that the number of clusters be prespecified, and assumes either a particular model of allele frequency correlations across clusters [12,13] or that allele frequencies are uncorrelated. The correlated frequencies model—the *F* model in [13]—supposes that the various clusters represent populations that have descended with genetic drift from a common ancestral population, so that alleles in different clusters have correlated frequencies due to shared ancestry. The uncorrelated frequencies model, on the other hand, is based on an assumption that allele frequencies are not expected to be similar across populations, and does not hypothesize an ancestral relationship among the clusters [12]. The reasoning underlying the correlated frequencies model is that for closely related populations, as measured by statistics such as *F_st_*, allele frequencies tend to be correlated. Including correlation in the population structure model typically gives STRUCTURE greater power to detect similar but distinct populations (Figure 2 of [13]).

**Figure 2:**
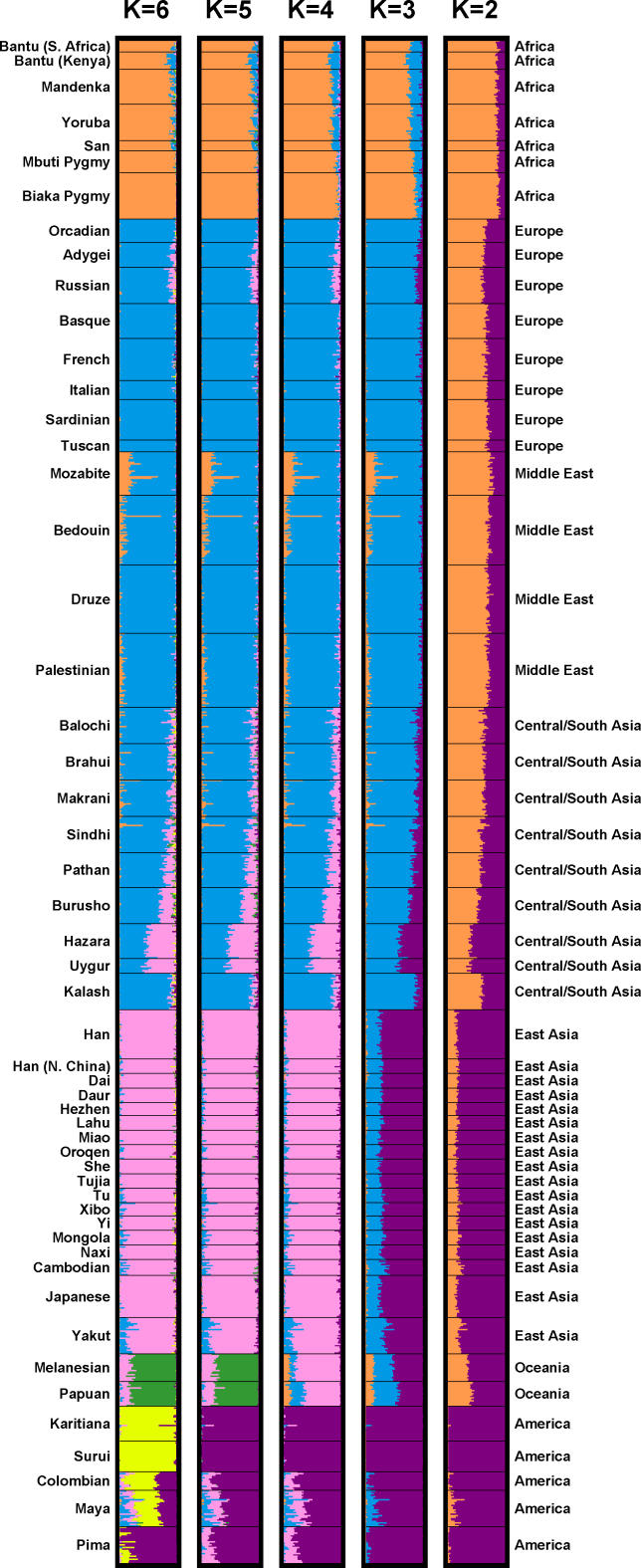
Inferred Population Structure Based on 1,048 Individuals and 993 Markers, Assuming Correlations among Allele Frequencies across Clusters Each individual is represented by a thin line partitioned into *K* colored segments that represent the individual's estimated membership fractions in *K* clusters. Each plot, produced with DISTRUCT [23], is based on the highest-likelihood run of ten runs: the two runs that were used in further analysis, and the eight runs described under “Cluster Analysis using STRUCTURE.” As in [3], four of ten runs with *K* = 3 separated a cluster corresponding to East Asia instead of one corresponding to Europe, the Middle East, and Central/South Asia. Two of ten runs with *K* = 5 separated Surui instead of Oceania. The highest-likelihood run of the ten runs with *K* = 6, shown in the figure, had a different pattern from the other nine runs (not shown). These other runs, instead of subdividing native Americans into two clusters, subdivided a cluster roughly similar to the Kalash cluster seen in [3], except with a less pronounced separation of the Kalash population. The clusteredness scores for the plots shown with *K* = 2, 3, 4, 5, and 6 are 0.50, 0.76, 0.84, 0.86, and 0.87, respectively.

A total of 367,220 runs of STRUCTURE were performed on subsets of a dataset consisting of 1,048 individuals from the Human Genome Diversity Project–Centre d'Etude du Polymorphisme Humain (HGDP-CEPH) Human Genome Diversity Panel [14] and 993 microsatellite and insertion/deletion polymorphisms. These runs utilized five choices for the number of clusters (two, three, four, five, and six), seven choices for the number of loci (ten, 20, 50, 100, 250, 500, and 993), four choices for the sample size (100, 250, 500, and 1,048), and two choices for the allele frequency correlation model (correlated and uncorrelated, as described by [12,13]). For each choice of the number of loci other than 993, runs were performed with each of ten prespecified sets of loci randomly selected from among the full set of markers, and for each choice of the sample size other than 1,048, runs were performed with each of 100 prespecified sets of individuals.

**Figure 3:**
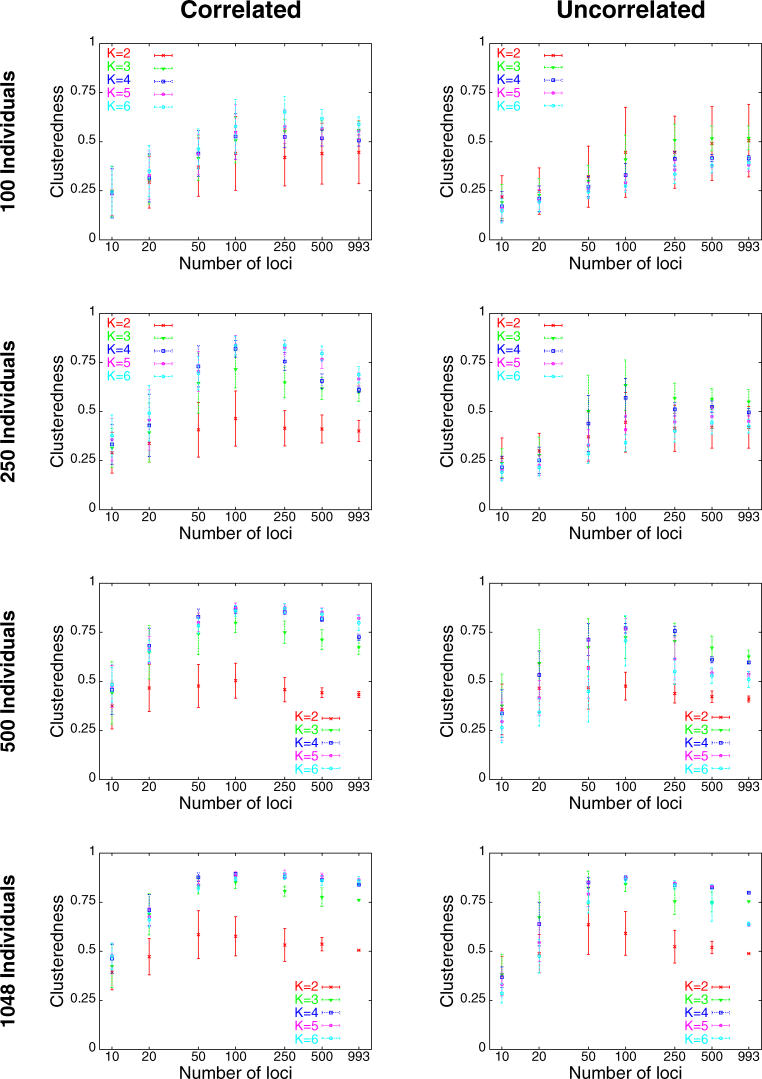
Mean Clusteredness versus Number of Loci Each point shows the mean clusteredness of 2,000 runs with the specified sample size and allele frequency correlation model: two replicates for each of ten sets of loci for each of 100 sets of individuals (for 1,048 individuals, it is the mean of 20 runs, as only one set of individuals was used; for 1,048 individuals and 993 loci, it is the mean of two runs, as only one set of loci was used). Error bars denote standard deviations. The *x*-axis is plotted on a logarithmic scale.

The 100 sets of individuals used were selected to have a wide range of levels of geographic dispersion (Figure 1), as measured by the dispersion statistic *A_n_* (see Materials and Methods). Because the sets all utilized the sampling locations of the diversity panel, their *A_n_* values were bounded by the minimal and maximal levels of dispersion possible in this sample. However, with a sample size of 100, the sets that had the lowest values of *A_n_*—and were therefore most uniformly distributed geographically—had comparable *A_n_* values to some sets of 100 points randomly chosen from the land area of the earth. For each collection of settings—the lists of individuals and loci, and the choices for the number of clusters and the allele frequency correlation model—two replicate STRUCTURE runs were performed. The “clusteredness” (see Materials and Methods) of the collection of estimated membership coefficients was then calculated for each of the 367,220 runs. This statistic measures the extent to which a randomly chosen individual is inferred to have ancestry in only one cluster (clusteredness = 1), with the other extreme being equal membership in all clusters (clusteredness = 0). Use of this statistic relies on the observation that when populations are unstructured or when insufficient data are used, STRUCTURE typically distributes the membership coefficients of all individuals evenly across clusters rather than assigning each individual a membership coefficient of one for one cluster (the same cluster for all individuals) and zero for all other clusters (see the top right plot in Figure 4 of [6] and the top left plot in Figure 6 of [9]).

**Figure 4:**
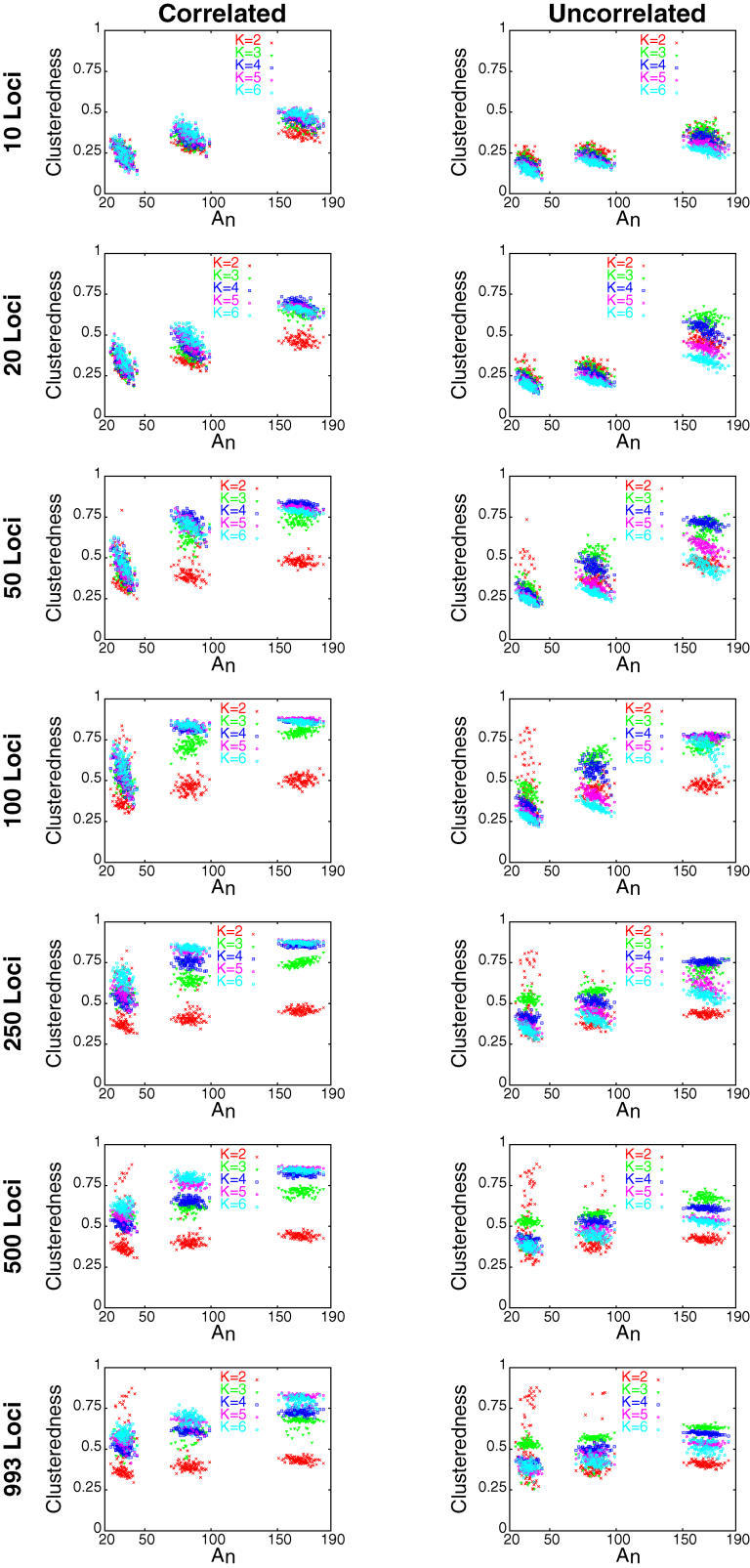
Mean Clusteredness versus Geographic Dispersion as Measured by *A_n_* Each point shows the mean clusteredness of 20 runs with the specified number of loci and allele frequency correlation model: two replicates for each of ten sets of loci (for 993 loci, it is the mean of two runs, as only one set of loci was used). From left to right, the three groups of points in each plot respectively represent sets of 100, 250, and 500 individuals.

**Figure 5:**
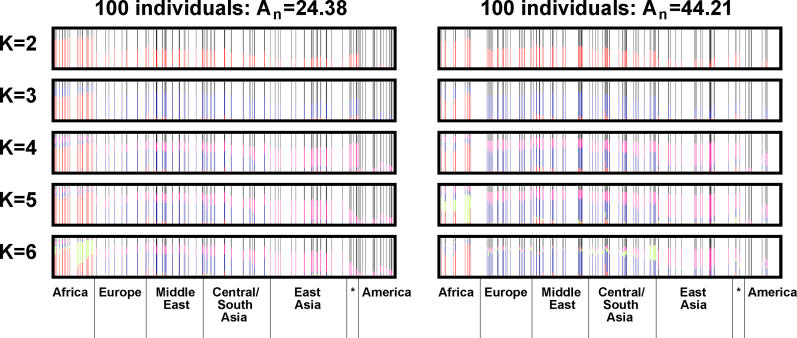
Inferred Population Structure Based on Two Different Sets of 100 Individuals, Using 993 Markers and the Correlated Allele Frequencies Model The two sets of 100 individuals represent extremes of the distribution of *A_n_:* the plots on the left are based on a more geographically random sample, and those on the right are based on a less random sample. Each plot is based on the higher-likelihood run among the two runs performed with the given combination of loci and individuals. In all plots, individuals and populations are in the same order as in Figure 2. Black vertical lines at the bottom of the figure separate populations from the different geographic regions described in [3], with the asterisk representing Oceania.

**Figure 6:**
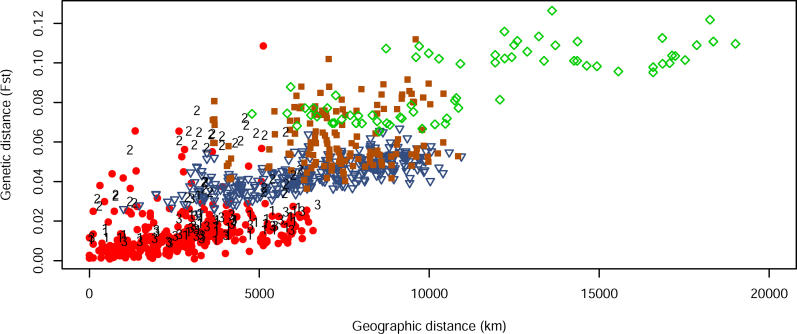
Genetic and Geographic Distance for Pairs of Populations Red circles indicate comparisons between pairs of populations with majority representation in the same cluster in the *K* = 5 plot of Figure 2; blue triangles indicate pairs with one population from Eurasia and one from East Asia; brown squares indicate pairs with one population from Africa and the other from Eurasia; and green diamonds indicate pairs with one population from East Asia and the other from either Oceania or America. Comparisons involving one of Hazara, Kalash, and Uygur and other populations from Eurasia or East Asia are marked 1, 2, and 3, respectively. No comparisons are shown between any of these three groups and any African population.

Representative estimates of the population structure based on the full dataset are shown in Figure 2. These estimates are quite similar to what was previously obtained using 377 loci [3], with the main difference being that the sixth cluster sometimes corresponds to a subdivision of native Americans into more northerly and more southerly populations rather than to a separation of the isolated Kalash population of Pakistan.

To examine the influence of the study design parameters on clusteredness, we separately considered each variable, holding the others constant. This analysis included linear regressions of clusteredness on each variable for each possible combination of values of the other variables. We also analyzed the full collection of runs to determine the relative contributions of the quantities considered to variability in clusteredness.

### Number of Loci

Holding the number of clusters, sample size, and allele frequency correlation model fixed, the general trend was that clusteredness was noticeably smaller for ten and 20 loci, and was larger for 50 or more loci (Figure 3). This was usually true regardless of the choice of the number of clusters, sample size, or correlation model. For 39 of 40 combinations of these three variables, the regression coefficient of the logarithm of the number of loci was significantly different from zero at the *p* < 0.001 level, indicating a noticeable effect of the number of loci on clusteredness (the 40th combination had *p* = 0.002). For all 40 combinations, the regression coefficient was positive, indicating an increase in clusteredness with increasing number of loci, and the mean coefficient of determination (*R* ^2^) across the 40 regressions equaled 0.454.

### Number of Clusters

When the number of loci, sample size, and correlation model were held constant, *K* = 2 (that is, two clusters) generally produced smaller clusteredness than did the larger values of *K* (Figures 3 and 4; Table 1). For the correlated allele frequencies model, *K* = 5 and *K* = 6 tended to have higher clusteredness than did *K* = 3 and *K* = 4, whereas the reverse was true for the uncorrelated model (Figure 4). This trend was reflected in the regression coefficients for *K:* with the correlated model, for 27 of 28 combinations of the number of loci and the sample size, the regression coefficient was positive, whereas it was positive for only 11 of 28 combinations with the uncorrelated model (Table 2). In 51 of 56 combinations, the regression coefficient was significantly different from zero at *p* < 0.001; 34 of these involved positive and 17 involved negative regression coefficients. Reflecting the general monotonic trend in clusteredness with *K* in the correlated model but not in the uncorrelated model, the average *R* ^2^ was larger across the 28 combinations with the correlated model (0.382) than it was for the 28 combinations with the uncorrelated model (0.147).

**Table 1:**
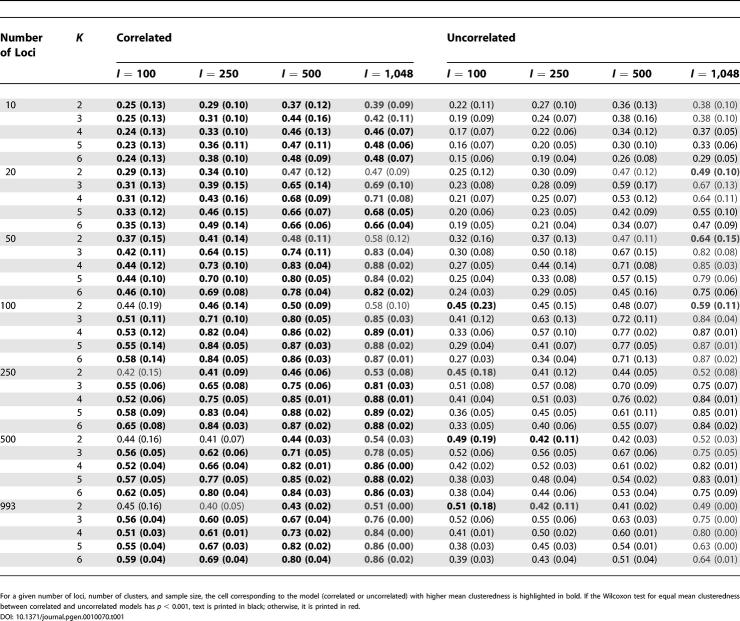
Clusteredness Mean and Standard Deviation for the Correlated and Uncorrelated Allele Frequency Models

**Table 2:**
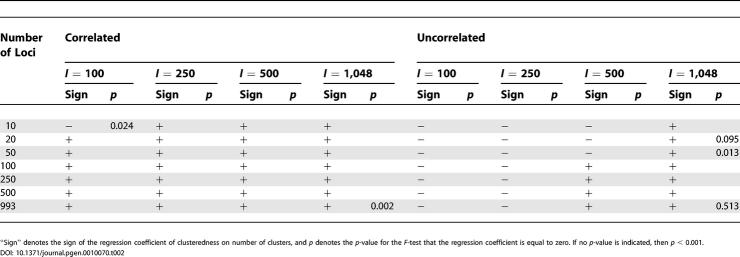
Influence of the Number of Clusters *K* on Clusteredness

### Sample Size

Holding the number of loci, number of clusters, and correlation model fixed, clusteredness was generally higher for the samples of size 250 and 500 than it was for the samples of size 100 (Figures 3 and 4; Table 1). For 65 of 70 combinations of the number of loci, the number of clusters, and the correlation model, the regression coefficient for sample size was both significantly different from zero at *p* < 0.001 and positive (Table 3). The five cases for which the regression coefficient was negative, not significantly different from zero at *p* < 0.001, or both all involved *K* = 2. The average *R* ^2^ across the 70 combinations equaled 0.511.

**Table 3:**
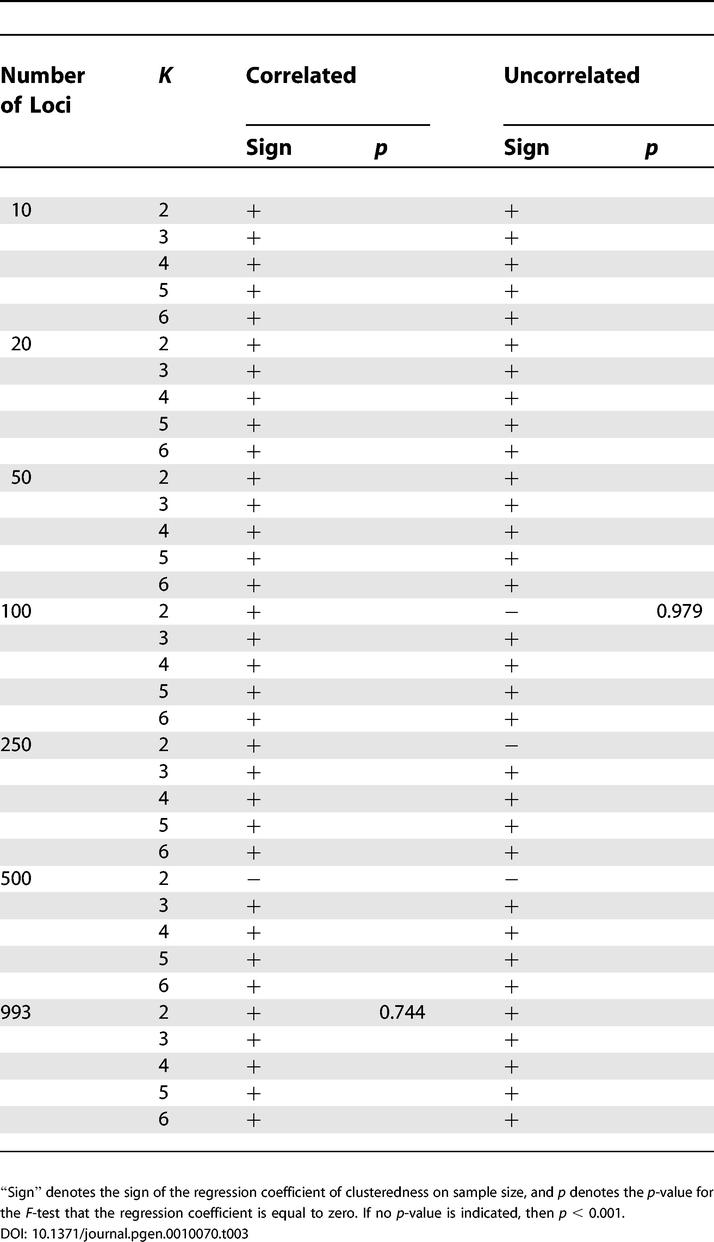
Influence of the Sample Size on Clusteredness

### Geographic Dispersion of Individuals

With the correlation model and the numbers of loci, clusters, and individuals held constant, the inferred population structure was generally similar for different values of *A_n_* (Figure 5, for example). Population structure estimates differed substantially for different values of *A_n_* mainly in situations where one but not the other dataset had a very small sample from one of the main clusters in the full dataset. For example, Oceania is well-represented and corresponds to a cluster for the more geographically random dataset in Figure 5 (left side), but is not well-represented and does not correspond to a cluster for the less random dataset (Figure 5, right side).

Often, geographic dispersion had a negative rather than a positive influence on clusteredness (see Figure 4), so that less uniformly distributed samples produced lower clusteredness. This effect was reflected in the regression coefficient for *A_n_,* which was negative for 174 of 210 combinations of the number of loci, sample size, number of clusters, and correlation model (Table 4). Of the 36 combinations with positive regression coefficients, 12 had regression coefficients that were significantly different from zero at *p* < 0.001. However, the decrease of clusteredness with increasing *A_n_* in the remaining 174 cases was often quite small; in 46 of these 174 cases, the regression coefficient was not significantly different from zero at *p* < 0.001, and the average *R* ^2^ across the 210 regressions was only 0.045.

**Table 4:**
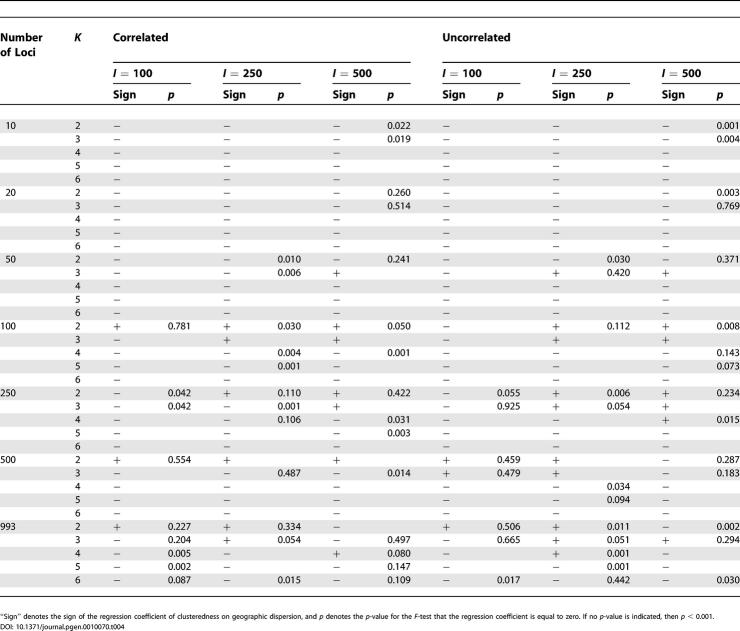
Influence of the Geographic Dispersion *A_n_* on Clusteredness

### Allele Frequency Correlation Model

With the numbers of loci, clusters, and individuals held constant, the correlation model had a noticeable influence on clusteredness, with the correlated model usually producing higher clusteredness than the uncorrelated model (see Figures 3 and 4; Table 1). This effect was generally seen regardless of the number of loci (Table 1). In 101 of 105 combinations in which the sample size was 100, 250, or 500, the Wilcoxon test for a difference in clusteredness under the correlated versus under the uncorrelated model was significant at *p* < 0.001. In 97 of these 101 combinations, the correlated model had higher mean clusteredness across runs than did the uncorrelated model. For 1,048 individuals, fewer runs were performed, and *p* < 0.001 for only 14 of 35 combinations; as with smaller sample sizes, however, in 32 of the 35 combinations with 1,048 individuals, clusteredness was greater for the correlated model. Considering all sample sizes, all nine cases in which clusteredness was smaller for the correlated model involved *K* = 2.

### Analysis of Variance of Clusteredness

With each sample size, considering all 122,000 STRUCTURE runs with the given sample size, the *R* ^2^ values for regressions of clusteredness on individual variables were greatest for the number of loci and the allele frequency correlation model, and smallest for the number of clusters and the geographic dispersion (Table 5). Combining all 367,220 runs, the sample size also produced an effect comparable to that seen for the number of loci and the correlation model, while the contributions of the number of clusters and the geographic dispersion remained smaller.

**Table 5:**
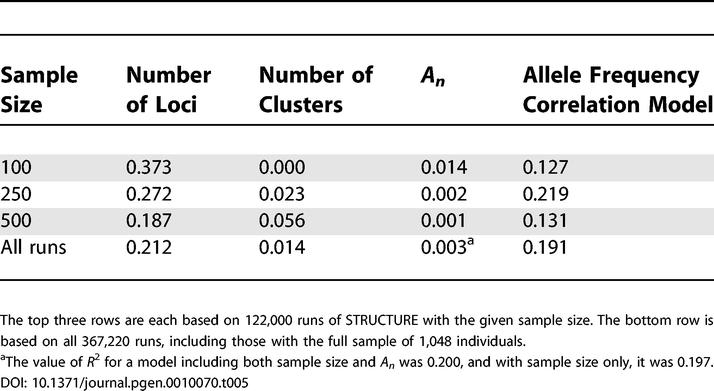
Values of *R* ^2^ for Regressions of Clusteredness on Study Design Variables

## Discussion

In this article, we have systematically analyzed the influence of five variables on the genetic clustering of individuals from genome-wide markers: number of loci, sample size, number of clusters, geographic dispersion of the sample, and assumptions about allele frequency correlation. Each of these variables was found to have an effect on clustering. Holding all other variables constant, geographic dispersion had a relatively modest effect on clusteredness, with a considerably smaller *R* ^2^ than number of loci, sample size, or number of clusters. Additionally, geographic dispersion was generally less consistent in the direction in which it affected clusteredness, although in contrast to what was expected based on the results of [10], samples with higher *A_n_* (that is, samples that were less geographically random) produced lower clusteredness more often than they produced higher clusteredness.

Unlike geographic dispersion, the number of loci and sample size both had strong direct relationships with clusteredness for nearly all combinations of the other variables. Excluding a few scenarios that utilized two clusters, the correlation model produced significantly greater clusteredness for nearly all combinations of the other variables, when a large number of STRUCTURE runs were performed. The number of clusters influences the way in which individual membership coefficients are distributed, but its effect on the clusteredness statistic was found to be smaller than that of the number of loci or the sample size. The effect of the number of clusters depended on the choice of correlation model: in the correlated model, clusteredness generally increased with *K,* whereas in the uncorrelated model, clusteredness was not monotonic in *K*.

Two main claims of Serre and Pääbo [10] merit direct comparison with our results. First, on the basis of STRUCTURE runs of two samples with 89 and 90 individuals, 20 loci, and the uncorrelated allele frequencies model, Serre and Pääbo argued that use of a sample with a more random geographic distribution led to reduced clusteredness. Although we were expecting to corroborate this observation, which was not based on the HGDP-CEPH Human Genome Diversity Panel sample studied here, our analysis under similar conditions did not support it. Moving across the range of *A_n_* for the 100 samples of size 100, when 20 loci were used with the uncorrelated model (Figure 4), there was a trend opposite to that expected, in that clusteredness decreased with increasing geographic nonrandomness: for a sample size of 100 and 20 loci, regression coefficients for *A_n_* were negative with *p* < 0.001 for each value of *K* and both correlation models (see Table 4). In other words, in a test similar to that performed by [10], the effect of reduced clusteredness with increasing geographic nonrandomness was not seen when 100 samples were studied, rather than two samples, as in [10].

Second, in three analyses with the uncorrelated allele frequencies model, each of which used 261 individuals, Serre and Pääbo observed a reduction in clusteredness compared with analyses using 261 individuals and the correlated model, and compared with analyses based on 1,066 individuals and either model. They attributed the different results in these scenarios to the use of the uncorrelated frequencies model. We found, however, that with either the correlated or the uncorrelated allele frequencies model, holding all other variables constant, when 100 samples of size 250 were considered, clusteredness differed for the samples of size of 250 compared with those of size 1,048 (Figure 3). Therefore, the difference in results obtained by [10] is likely to derive from a combination of both the difference in models and the difference in sample size.

Even if the frequency correlation model actually provided the sole explanation for the weaker clustering in their analysis, we question the basis for assuming that allele frequencies are uncorrelated across populations. Allele frequencies should be expected to be correlated, on the basis of the shared descent of all human populations from the same set of ancestral groups. Clearly, as has been shown in simulations [13], the choice of correlation model has a substantial influence on clustering results (Figures 3 and 4; Table 1). However, as the correlated and uncorrelated models should only be expected to produce different results if data contain a high level of correlation—which is taken into account by the correlated model but not by the uncorrelated model—it is precisely when allele frequencies have strong correlations across populations that the two models will produce different results. Thus, the high correlation coefficients we have estimated for allele frequencies ([9]; Table 6) both explain the difference in results between the correlated and uncorrelated models, and suggest that the correlated model, which we used in [3] and in Figure 2, provides a more appropriate model for human genetic variation.

**Table 6:**
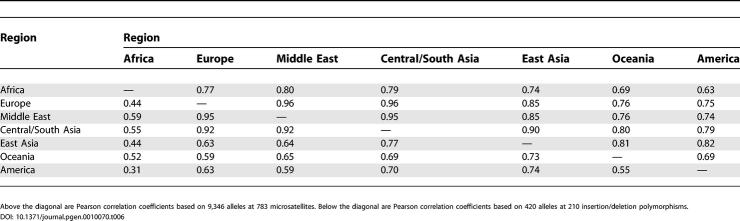
Correlation Coefficients of Allele Frequencies

In summary, the observation of [10] of stronger clustering with increased geographic nonrandomness was not seen in our analysis of a larger number of samples. Additionally, geographic dispersion was seen to be the least influential of the five study design variables that we considered. By using fewer loci and individuals in their various tests, and by assuming an uncorrelated allele frequencies model, Serre and Pääbo chose study design parameters in such a way that clustering was less pronounced than had been previously observed. In no way does this alter the fact that when a sufficiently large sample and number of loci are used, together with the more appropriate correlated allele frequencies model, individuals do cluster into populations that correspond largely to geographic regions. Indeed, the observation of essentially the same clusters with a larger dataset further supports the robustness of our original analysis.

### Clines or Clusters?

Serre and Pääbo [10] argue that human genetic diversity consists of clines of variation in allele frequencies. We agree and had commented on this issue in our original paper ([3], p. 2382): “In several populations, individuals had partial membership in multiple clusters, with similar membership coefficients for most individuals. These populations might reflect continuous gradations across regions or admixture of neighboring groups.” At the same time, we find that human genetic diversity consists not only of clines, but also of clusters, which STRUCTURE observes to be repeatable and robust.

How can these seemingly discordant perspectives on human genetic diversity be reconciled? Figure 6 shows a plot of genetic distance and geographic distance for pairs of populations. To illustrate the effects of moving continuously across geographical space, only pairs from within clusters or from geographically adjacent clusters are shown. That is, for the five clusters with *K* = 5 in Figure 2 of the present study and in Figure 1 of [3]—corresponding to Africa, Eurasia (Europe, Middle East, and Central/South Asia), East Asia, Oceania, and the Americas—an intercluster population pair is plotted only if it includes one population from Africa and one from Eurasia, one from Eurasia and one from East Asia, or one from East Asia and one from Oceania or the Americas.

For population pairs from the same cluster, as geographic distance increases, genetic distance increases in a linear manner, consistent with a clinal population structure. However, for pairs from different clusters, genetic distance is generally larger than that between intracluster pairs that have the same geographic distance. For example, genetic distances for population pairs with one population in Eurasia and the other in East Asia are greater than those for pairs at equivalent geographic distance within Eurasia or within East Asia. Loosely speaking, it is these small discontinuous jumps in genetic distance—across oceans, the Himalayas, and the Sahara—that provide the basis for the ability of STRUCTURE to identify clusters that correspond to geographic regions.

Two exceptions to the pattern include the Hazara and Uygur populations, from Pakistan and western China, respectively, whose genetic distances scale continuously with geographic distance both for populations in Eurasia and for those in East Asia. These populations were evenly split across the clusters corresponding to Eurasia and East Asia, and thus, unlike most other populations, they do not reflect a discontinuous jump in genetic distance with geographic distance. Finally, a third population of interest in the plot is the Kalash population (of Pakistan), whose genetic distances to other populations are large at all geographic distances, illustrating the distinctiveness of the group as the only member of its own genetic cluster in some STRUCTURE analyses with *K* = 6 [3].

Excluding points that involve Hazara, Kalash, or Uygur, a linear regression on geographic distance for the points in Figure 6 has *R* ^2^ = 0.690. When an additional binary variable *B* is added—equaling one if an ocean, the Himalayas, or the Sahara must be crossed to travel between two populations, and zero otherwise—*R* ^2^ increases to 0.729. The regression equation is *F_st_* = 0.0032 + 0.0049*D* + 0.0153*B*, where *D* is distance in thousands of kilometers. By dividing the regression coefficients for *B* and *D,* it can be observed that crossing one of the barriers adds an equivalent amount of genetic distance as traveling approximately 3,100 km on the same side of the barrier. The effect of a barrier is to add 0.0153 to *F_st_* beyond the value predicted by geographic distance alone. As 0.0153 is not a large value of genetic distance, and because the addition of the *B* term produces only a modest increase in *R* ^2^, the discontinuities that give rise to genetic clusters—as we have stated previously [3]—constitute a relatively small fraction of human genetic variation.

Our evidence for clustering should not be taken as evidence of our support of any particular concept of “biological race.” In general, representations of human genetic diversity are evaluated based on their ability to facilitate further research into such topics as human evolutionary history and the identification of medically important genotypes that vary in frequency across populations. Both clines and clusters are among the constructs that meet this standard of usefulness: for example, clines of allele frequency variation have proven important for inference about the genetic history of Europe [15], and clusters have been shown to be valuable for avoidance of the false positive associations that result from population structure in genetic association studies [16]. The arguments about the existence or nonexistence of “biological races” in the absence of a specific context are largely orthogonal to the question of scientific utility, and they should not obscure the fact that, ultimately, the primary goals for studies of genetic variation in humans are to make inferences about human evolutionary history, human biology, and the genetic causes of disease.

## Materials and Methods

### Data.

The dataset analyzed here consists of 1,048 individuals from the HGDP-CEPH Human Genome Diversity Panel [14]. Each individual was genotyped by the Mammalian Genotyping Service for 993 polymorphisms spread across all 22 autosomes: 783 microsatellites (with 3.7% missing data) and 210 insertion/deletion markers (with 7.7% missing data). Of these loci, 377 of the microsatellites were previously studied by [3] in most of the individuals analyzed here. The remaining microsatellites were drawn from Marshfield Screening Sets #13 and #52 [17], and the insertion/deletion markers were drawn from those studied by [18]. All 783 of the microsatellites were previously studied by [11].

The set of individuals used here differs slightly from that studied by [3]. It corresponds exactly to the set in [11], with two alterations. First, 21 Surui individuals excluded by [11] are included here, and second, eight individuals grouped into the southwestern Bantu and southeastern Bantu populations in [11] are grouped here as a single population labeled Bantu (southern Africa). Thus, we analyzed 53 populations.

### Geographic dispersion.

The geographic dispersion of a set of *n* points on a sphere can be measured by the statistic


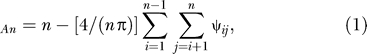


where ψ*_ij_* is the angle between the *i*th and *j*th points measured at the center of the sphere. The quantity *A_n_* is a test statistic for the null hypothesis that the *n* points are uniformly distributed on the sphere (p. 149 of [19]). Larger values of *A_n_* indicate sets of points that are less uniformly distributed. To evaluate ψ*_ij_* for a pair of points *i* and *j,* rectangular coordinates (*x*, *y*, *z*) are obtained from (latitude, longitude) coordinates (*a*, *b*) using (*x_i_*, *y_i_*, *z_i_*) = (cos(*a_i_*) cos(*b_i_*), cos(*a_i_*) sin(*b_i_*), sin(*a_i_*)) and (*x_j_*, y*_j_*, *z_j_*) = (cos(*a_j_*) cos(*b_j_*), cos(*a_j_*) sin(*b_j_*), sin(*a_j_*)). By the law of cosines,


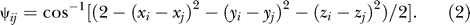


Method 1 of [20] was used to generate the rectangular coordinates for random points uniformly distributed on the sphere. For each sample size (*n* = 100, 250, or 500), 100,000 sets of points were considered in obtaining the distribution of *A_n_*.

To determine the distribution of *A_n_* for sets of points uniformly distributed on the land area of the earth, 4,210 lattice points on land were identified for a lattice of 200 longitudes and 79 latitudes on the earth's surface (Figure 5 of [11]). From these points, for each sample size (*n* = 100, 250, or 500), 100,000 sets of points were drawn (with replacement), and *A_n_* was calculated for each set.

To obtain the distribution of *A_n_* for sets of points randomly chosen from the dataset, for each sample size (*n* = 100, 250, or 500), 100,000 random subsets of the 1,048 individuals were selected (without replacement), and *A_n_* was computed for each subset. Latitude and longitude coordinates were taken from Supplementary Table 1 of [14]. In cases where latitudes and longitudes were given as ranges, the centroid of the specified region was calculated, with the longitude being the average of the endpoints of the range and the latitude being the inverse sine of the average of the sines of the endpoints of the range. Of the 100,000 random subsets of individuals, the 100 sets located at quantiles *c* + 1/2 with respect to the distribution of *A_n_* were utilized in further analyses, where *c* ranged over integers from zero to 99.

### Clusteredness.

To measure the average “clusteredness” of individuals, or the extent to which individuals were estimated to belong to a single cluster rather than to a combination of clusters, we computed for each STRUCTURE run the quantity


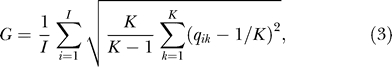


where *q_ik_* denotes the estimated membership coefficient for the *i*th individual in the *k*th cluster, *I* denotes the total number of individuals, and *K* denotes the total number of clusters. The factor *K*/(*K −* 1) was included so that a change in *K* would not produce a systematic change in clusteredness.

### Cluster analysis using STRUCTURE.

All runs of the STRUCTURE program [12] employed for analyzing the study design variables utilized 1,000 iterations after a burn-in period of 5,000 iterations. To evaluate whether this length was sufficient for convergence, we performed longer runs, all with a burn-in period of 5,000, and we compared results based on later iterations with those of the first 1,000 iterations after the burn-in. For each of *K* = 2, 3, 4, 5, and 6, eight runs were performed using the full dataset and the correlated allele frequencies model. Estimates of membership coefficients were separately obtained using the first 1,000 iterations after completion of the burn-in, iterations 15,001–20,000 after the burn-in, and iterations 45,001–50,000. Using a symmetric similarity coefficient [21], each of these three stages in each run was compared to each stage in the other seven runs with the same value of *K,* as well as to the other two stages from the same run. In all cases except for one of the runs with *K* = 6, similarity scores were 0.96 or greater, indicating that membership coefficient estimates were nearly identical both for different runs with the same *K* as well as for the three stages of the same run. Thus, it was determined that estimates would not be substantially different if runs longer than 1,000 iterations after a burn-in period of 5,000 were used. For each *K,* the results obtained from the eight runs at 1,000 iterations after completion of the burn-in were among the ten runs considered in choosing the highest-likelihood runs to display in Figure 2.

### Statistical tests.

Linear regression was used to test the influence of study design variables on clusteredness. To control for the effects of the other variables, each regression utilized only STRUCTURE runs in which variables other than the one being tested were held constant. For example, to examine the influence of the number of clusters on clusteredness, 56 separate regressions were performed, one for each combination of the number of loci (seven possibilities), the sample size (four possibilities), and the allele frequency correlation model (two possibilities). Similarly, 40 regressions of clusteredness on the base-10 logarithm of the number of loci were performed, as were 70 regressions of clusteredness on sample size and 210 regressions of clusteredness on *A_n_*. Note that in the case of *A_n_,* since there was no variability in *A_n_* across different runs with the full 1,048 individuals, the number of regressions reflects seven choices for the number of loci, five choices for the number of clusters, two choices for the allele frequency correlation model, and only three choices for the sample size. For each regression, the *F*-test was used to test the null hypothesis that the regression coefficient for the dependent variable equaled zero.

In the case of the allele frequency correlation model, the runs with the correlated and uncorrelated models were compared using the Wilcoxon two-sample test instead of with linear regression. Because there were seven numbers of loci, five numbers of clusters, and four numbers of individuals, 140 separate tests were performed.

For each sample size, regressions of clusteredness on individual variables were also performed using all 122,000 runs with the given sample size. Additional regressions were also performed using all 367,220 runs. These regressions used the base-10 logarithm of the number of loci.

### Genetic and geographic distance.

For the comparison of genetic and geographic distance, calculations were performed as in [11], using *F_st_* for genetic distance—computed as *F_st_* = −ln(1 − θ), with the estimate of θ taken from equation 5.12 of [22]—and waypoint routes avoiding large bodies of water for geographic distance. A slight difference from the analysis in [11] was that the great circle distance for a pair of points *i* and *j* was computed using *r*ψ*_ij_* where *r* is the radius of the earth (6,371 km) and ψ*_ij_* is measured in radians, rather than with equation 1 of [11]. Only the microsatellite data were used for this analysis, and the Karitiana, Maya, and Surui were omitted from the comparisons: Maya due to likely admixture [3], and Karitiana and Surui to keep the ranges of the axes in the plot small enough for the patterns of interest to be visible. See [11] for additional related plots.

## Abbreviations

HGDP-CEPH: Human Genome Diversity Project–Centre d'Etude du Polymorphisme Humain
